# An assessment of implementation and effectiveness of mass drug administration for prevention and control of schistosomiasis and soil-transmitted helminths in selected southern Malawi districts

**DOI:** 10.1186/s12913-022-07925-3

**Published:** 2022-04-19

**Authors:** Peter Makaula, Sekeleghe Amos Kayuni, Kondwani Chidzammbuyo Mamba, Grace Bongololo, Mathias Funsanani, Janelisa Musaya, Lazarus Tito Juziwelo, Peter Furu

**Affiliations:** 1 Research for Health Environment and Development (RHED), P.O. Box 345, Mangochi, Malawi; 2 Medical Aid Society of Malawi (MASM) Medi Clinics Limited, Area 12 Medi Clinic, P.O. Box 31659, Lilongwe, 3 Malawi; 3 Malawi Liverpool Wellcome Clinical Research Programme, Private Bag 30096, Chichiri, Blantyre, 3 Malawi; 4 Liverpool School of Tropical Medicine, Pembroke Place, Liverpool, L3 5QA UK; 5 Mangochi District Council, District Health Office, P.O. Box 42, Mangochi, Malawi; 6 Department of Pathology, Kamuzu University of Health Sciences, Private Bag 360, Chichiri, Blantyre, 3 Malawi; 7 Ministry of Health, Community Health Sciences Unit, National Schistosomiasis and Soil-Transmitted Helminths (STH) Control Programme (NSCP), Private Bag 65, Lilongwe, Malawi; 8 Department of Public Health, Global Health Section, University of Copenhagen, P. O Box 2099, 1014 Copenhagen K, Denmark

**Keywords:** Mass drug administration, Neglected tropical diseases, Schistosomiasis, Soil-transmitted helminths

## Abstract

**Background:**

Mass drug administration (MDA) is one of the key interventions recommended by WHO for prevention and control of neglected tropical diseases (NTD). In Malawi, MDA is widely carried out annually since 2009 for prevention and control of schistosomiasis and soil-transmitted helminths (STH). No study has been carried out to assess effectiveness of the MDA approach and to document perceptions of health providers and beneficiaries regarding use of MDA. This study was done to understand how well MDA is being implemented and to identify opportunities for improvement in MDA delivery in Malawi.

**Methods:**

Designed as a cross-sectional and multi-methods research, the study was carried out in three southern Malawi districts of Chiradzulu, Mangochi and Zomba. In each district, four health centres and 16 villages were randomly selected to participate. A mixed-methods approach to data collection focusing on quantitative data for coverage and knowledge, attitudes and practices assessments; and qualitative data for assessing perceptions of health providers and beneficiaries regarding MDA was used. Quantitative data were processed and analyzed using IBM SPSS software version 26 while qualitative data were analysed using NVivo 12 for Windows.

**Results:**

Knowledge levels about schistosomiasis and STH in the districts varied according to disease aspects asked about. Majority are more knowledgeable about what schistosomiasis is (78%) and whether STH are treatable with drugs (97%); with least knowledgeable about the organism that transmits schistosomiasis (18%), types of schistosomiasis (11%) and what causes STH (20%). In 2018 and 2019 the districts registered high coverage rates for praziquantel and albendazole using community-based MDA (73–100%) and using school-based MDA (75–91%). Both the health authorities and community members perceived the MDA approach as good because it brings treatment closer to people.

**Conclusion:**

With the high MDA coverage obtained in communities and schools, the effectiveness of MDA in the target districts is satisfactory. There are, however, several challenges including disproportionate knowledge levels, which are hampering progress towards attainment of the 2030 global NTD goals. There is a need for promotion of community participation and partnerships as well as implementation of other recommended interventions for sustainable prevention and control of schistosomiasis and STH.

**Supplementary Information:**

The online version contains supplementary material available at 10.1186/s12913-022-07925-3.

## Background

Neglected tropical diseases (NTD) represent a diverse group of human and zoonotic diseases whose health and economic burden fall most heavily on the poorest people and communities [1]. These include diseases such as soil-transmitted helminthiases (STH), lymphatic filariasis (LF), onchocerciasis, schistosomiasis, dengue and rabies among the 20 listed diseases [1]. Globally, at least 1.7 billion people in 185 countries are affected by NTD and require mass or individual treatment and care to minimize the burden. Of these, 1.1 billion (65%) are in low- and middle-income countries which are affected by at least five NTD [1]. Access to health interventions continues to be a major challenge for many people in rural settings in low- and middle-income countries of the world including sub-Saharan Africa where health systems are weak and suffer from inadequate mechanisms for delivering vital health services to those in most need [2, 3]. Preventive chemotherapy using mass drug administration (MDA), case-management, vector management, environmental improvement measures and health promotion are the key interventions recommended by World Health Organization (WHO) for prevention and control of NTD [4].

In Malawi, most water bodies (20% of the country) are considered to be potential transmission sites for schistosomiasis [5] and schistosomiasis and STH, are some of the NTD that require special emphasis due to their considerable public health significance. These infections are highly prevalent amongst school aged children (SAC) as well as adults in most parts of the country. Various surveys carried across the country dating from 1994 to 2018 show that schistosomiasis and STH are major public health problems with prevalence ranges of between 50.0–94.9% for urinary schistosomiasis caused by *Schistosoma haematobium*, 34.0–67.0% for intestinal schistosomiasis caused by *Schistosoma mansoni* and 0.3–3.9% for STH mainly *Ascaris lumbricoides* (roundworms) and *Ancylostoma* sp. (hookworms) [5–17]. MDA is one of the community-based interventions, which has been widely carried out annually since 2009 [18]. During annual MDA campaigns, it is the health workers, mostly the community-based Health Surveillance Assistants (HSA) who are responsible for the distribution of drugs. Over the years of MDA implementation in Malawi, community coverage has been high at 87.0% (range: 51.5–95.0%) for praziquantel and 82.0% (range: 30.6–92.3% for albendazole due to donor support by the Schistosomiasis Control Initiative Foundation [18]. However, maintaining these high coverage rates pose a sustainability challenge for the MDA deliveries once the donor support comes to the end. Since the start of delivery of MDA in Malawi in 2009, no study has been carried out to assess the effectiveness of MDA on prevention and control of schistosomiasis and STH. This study was carried out in selected districts of Malawi to gain new knowledge and understanding of how well MDA is being implemented and to identify opportunities for improvement in MDA delivery.

## Methods

### Study design

The study was designed as a cross-sectional and multi-methods study to assess effectiveness, examine perceived strengths and weaknesses, successes and failures, as well as health providers’ and beneficiaries’ perspectives of implementing the MDA strategy for prevention and control of selected NTD in selected Malawi districts.

### Study setting

Malawi is a landlocked country in South-eastern Africa with a 2020 projected population of 20,119,830 inhabitants [19]. The Malawi Government is the main public provider for health care services providing free health care services at the point of delivery close to 60% of all health care services through the Ministry of Health (MOH) [20]. The primary health strategy for the MOH is to ensure attainment of the universal health coverage through the implementation of the essential health package (EHP) programme [21]. The EHP programme addresses the major causes of morbidity and mortality among the general population and focuses particularly on medical conditions and service gaps that disproportionately affect the rural poor. The EHP has identified the prevention and control of schistosomiasis and STH as one of its priority areas toward attainment of the universal health coverage. Prevention and control of NTD in Malawi benefits from the existence of a national plan of action for NTD [5] and its incorporation in the overall Ministry of Health strategic plan [20]. Additionally, in the Malawi context, disease control takes advantage of current political will, well established partnerships, the occurrence of a defined structure for managing NTD spanning from national to district levels including the presence of skilled HSA and the availability of volunteers in the communities [20, 22].

### Study areas

**Fig. 1:**
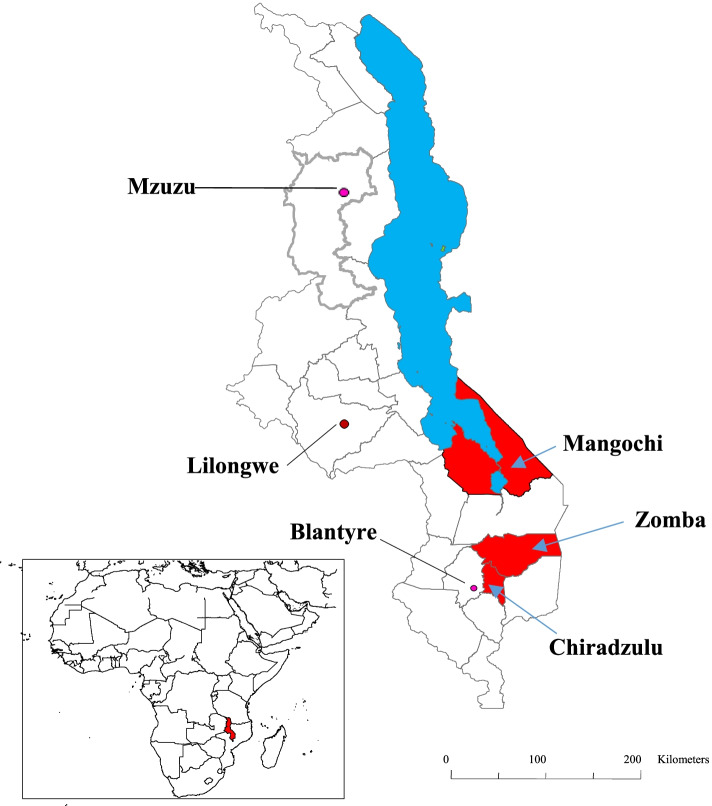
Locations of the study districts of Chiradzulu, Mangochi and Zomba (in red), Lake Malawi (in blue), major cities of Mzuzu, Lilongwe and Blantyre and the location of Malawi in Africa (red in the inset) (Source: Authors’ own map [23])

Three southern Malawi districts of Chiradzulu, Mangochi and Zomba (Fig. 1) were selected purposively based on their level of co-endemicity with the prioritized NTD of schistosomiasis and STH. Other study criteria included districts’ distinct topographies with both inland and lowland communities, which may have significant impact on the ecology and consequently on the transmission dynamics and prevalence of schistosomiasis and STH.

These districts are also amongst the high transmission areas with considerable prevalence of infection for both schistosomiasis and STH in Malawi [5–17]. The selection process for study sites involved consultations with the Director of Preventive Health Services and the National Schistosomiasis and STH Control Programme (NSCP) Manager. Selection of health centres and villages involved consulting respective District Health Management Team members in the target districts. From an exhaustive list of functioning health centres located in the three target districts, four health centres and 16 villages per district were randomly selected to participate in the study. Comparative socio-economic, demographic and health indicators for the three study districts and for Malawi as a whole are presented in Table 1.

**Table 1:** Comparative socio-economic, demographic and health indicators for the three study districts and for Malawi

Indicator	Chiradzulu	Mangochi	Zomba	Malawi
Population size/Density^a^	446,521/424	1,177,300/168	909,107/316	20,119,830/186
Predominant religion^a^	Christian (87.7%)	Islam (70.3%)	Christian (76.8%)	Christian (79.9%)
Level of literacy among women/men^a^	77.8%/93.3%	57.0%/71.1%	77.9%/87.7%	72.1%/82.9%
% of population with improved sanitation facilities^a^	57.0%	62.0%	60.0%	63.0%
Infant mortality rate^b^	45/1000	37/1000	80/1000	42/1000
Fertility rate^b^	5.1	5.9	5.4	4.4
Maternal mortality rate^c^	64/100,000	400/100,000	210/100,000	439/100,000
% of population with safe water^c^	73.0%	74.0%	82.0%	67.0%
No. of health workers (doctors/nurses/CHW per 100,000 population)^d^	0.9/30.0/46.1	0.4/17.9/40.9	0.9/26.5/65.9	1.9/33.7/76.2
No. of health facilities per 100,000 population^d^	3.6	3.6	4.1	4.7
Gross Domestic Product % of population under the poverty line (1US Dollar)^e^	65.4%	69.8%	67.5%	65.3%

Sources: ^a^Malawi Census Report, 2018 [19]

^b^Malawi Demographic and Health Survey, 2017 [24]

^c^District Social Economic Profiles for Chiradzulu, 2017 [25], Mangochi, 2017 [26] and Zomba, 2017 [27]

^d^Health Sector Strategic Plan II, 2017 [20]

^e^Malawi Growth and Development Strategy III, 2017 [28]

### MDA services, process and delivery

Mass drug administration is a means of delivering safe and inexpensive medicines based on the principles of preventive chemotherapy, where populations or sub-populations are offered treatment without prior individual diagnosis [4, 29–31]. The NSCP has since 2009 been organizing annual MDA campaigns in every district in order to distribute praziquantel and albendazole drugs against schistosomiasis and STH respectively. In every district, the campaigns are implemented by a multi-sectoral team led by NTD and School Health and Nutrition Coordinators from the Ministries of Health, and Education respectively. At community level, distribution of drugs is done by HSA with help from community volunteers in villages and teachers in schools. Supervision and monitoring of MDA delivery are done by health workers from the district and health centres. During these campaigns, it is mainly the school children aged between 5 and 15 who are targeted and prioritized although adults above 15 years also receive treatment. Implementation of MDA in Malawi follows the process and guidelines as laid down by WHO [4, 29–31].

### Study population and sampling

The 2020 projection of total population living in the 48 sampled villages was 53,954 of which 52.4% were female and 47.6% were male. Of the total population 15,401 (28.6%) were from Chiradzulu [25], 27,026 (50.2%) were from Mangochi [26] and 11,444 (21.2%) were from Zomba [27]. The study population comprised the District NTD Coordinators, Pharmacy Technicians, representatives of implementation partners involved in delivery of MDA for schistosomiasis and STH, officers in-charge (clinicians or nurses), Senior HSA, community leaders and adult community members (above age 14 years).

Sampling techniques deployed at district level included purposive selection of key informants namely, the District NTD Coordinators, Pharmacy Technicians, and representatives of some of the partners involved in delivery of MDA for schistosomiasis and STH in each district for face-to-face in-depth interviews. At health centre level, clinicians or nurses in-charge or their representatives, and Senior HSA were purposively selected to participate in the study. At community level, we purposively selected responsible HSA and community leaders from the targeted villages while taking into consideration their diverse gender and roles. In addition, to obtain a varied community representation and a detailed impression of community perceptions, we randomly invited different homogenous groups of eight to ten people from selected villages to participate in a focus group discussion (FGD). Lastly, in every village a predetermined number of households were randomly selected to participate in a face-to-face questionnaire-based knowledge, attitudes and practices survey. Any household member above 14 years available during the time of the visit was invited to participate in the survey while ensuring gender balance.

### Data collection

The study employed a mixed-methods approach to data collection focusing on quantitative data for effectiveness and qualitative data for assessing perceptions of health providers and beneficiaries, and evaluating processes regarding MDA intervention. Data were collected at district, health centre and village levels in all study districts in April, 2020 by ten graduate research assistants (five male and five female) who were specifically recruited based on their prior knowledge and skills in the research field. The research assistants were initially trained and later engaged in data collection from the involved health professionals, implementation partners, community leaders and beneficiaries using eight data collection instruments previously used by the research group in a 2010 multi-country study [23] and another 2016 study [32]. The instruments consisted of a survey questionnaire programmed in tablets and was administered to adult household representatives in their homes at community level for determining respondents’ knowledge, attitudes and practices regarding prevention and control of schistosomiasis and STH, and delivery of MDA. Interview guides were used to conduct in-depth interviews with professionals at district and health centre levels, implementation partners at district level in their respective workplaces, HSA and volunteers in villages at community level about their perceptions on benefits, critical factors and to evaluate the processes used during MDA intervention delivery. Moreover, schistosomiasis and STH treatment records for preceding years of 2018 and 2019 were obtained from health services and reviewed using checklists to establish MDA coverage data at district, health centre and village levels. Focus group discussion guides were used to conduct group interviews with beneficiaries about their perceptions on using the MDA intervention and benefits. An additional file shows the instruments that were used during data collection [see Additional file 1]. The interviews and focus group discussions lasted between 30 and 60 min and involved the participants and research assistants only. All the proceedings of the key informant in-depth interviews and focus group discussions were recorded using digital audio recorders. Using these tools data were collected in three districts comprising totals of 12 health centres and 48 villages. Finally, document reviews were carried out in order to get an insight on the national prescription of health policy, priority health issues, strategy and effectiveness of delivery of MDA, availability of resources for MDA and the existing challenges and opportunities. Table 2 summarizes the methods, purposes, levels and quantities of data collected in the study.

**Table 2:** Methods, purposes, levels and amount of data collected in the study

Methods	Purpose of data collected	Data collection - levels and numbers collected					Totals
		District	Implementation partners	Health Centre	Village	Household	
1. Questionnaire	Knowledge, attitudes and practices	–	–	–	–	379	379
2. In-depth interviews	Process/Perceptions/Benefits/Critical factors	6	3	12	41	–	62
3. Checklists/Observations	Coverage/Disease burden	6	–	12	–	–	18
4. Health Management and Information System	Coverage/Disease burden	3	–	–	–	–	3
5. Focus group discussions	Perceptions/Benefits/Critical factors	–	–	–	12	–	12
6. Document reviews	Policies/Processes/Priorities/Resources/Challenges/Opportunities	6	–	–	–	–	6

### Data management and analysis

Quantitative data collected through survey questionnaire was programmed in tablets using the Secure Data Kit [33]. Questionnaire and checklists data were processed and analyzed using IBM Statistical Package for Social Sciences (SPSS) software version 26. Analysis involved calculation of percentages, tabulations and frequencies to estimate MDA coverage. Knowledge which had closed questions was measured using aggregated percentages while attitudes and practices were measured using percentages derived from a five point Likert scale from which respondents indicated their extent of agreement or disagreement with statements in order to rate their responses to evaluative questions. Qualitative data consisted of textual and audio data, including translated transcripts of key informant in-depth interviews, transcripts of focus group discussions, field notes on observations and other intervention-specific insights, notes and reports from meetings. Qualitative content analysis of the data was done using NVivo 12 for Windows (QSR International). The data were independently scrutinized by two qualitative researchers through reading and re-reading to look for patterns and important issues. This was followed by manual identification and sorting into a codebook from which meaningful groups of codes were organized to come up with categories. The categories were further collapsed to form initial themes which were further refined, defined and interpreted [34]. For triangulation purposes, these processes were also repeated by a Social Science researcher by randomly selecting three audio recordings and four field notes scripts. We used the Consolidated Framework for Implementation Research [35] for organization and reporting of actionable findings about contextual and intervention barriers and facilitators affecting MDA implementation outcomes in the study districts.

## Results

### Assessment of knowledge, attitudes and practices regarding schistosomiasis and STH

The survey on knowledge, attitudes and practices levels regarding schistosomiasis and STH reached a total of 379 respondents comprising 268 (70.7%) female and 111 (29.3%) male drawn from the three districts of Chiradzulu, Mangochi and Zomba (Fig. 1). The mean age of the respondents was 40.7 years ranged between 16 and 89 years and majority (93.9%) of them resided in rural areas. Table 3 summarizes distribution of the socio-economic characteristics of respondents across the study districts.

**Table 3:** Socio-economic characteristics of survey respondents by districts

Characteristic	Number (%) of respondents who participated in the survey			
	Chiradzulu	Mangochi	Zomba	Totals
***1. No. of respondents***	126 (33.3)	129 (34)	124 (32.7)	379 (100)
***2. Sex***				
Female	91 (72.2)	92 (71.3)	85 (68.5)	268 (70.7)
Male	35 (27.8)	37 (28.7)	39 (31.5)	111 (29.3)
***3. Age (in years)***				
Mean	42.5	36.9	42.6	40.7
Range	18–89	18–87	16–81	16–89
***4. Location***				
Urban	21 (16.7)	–	1 (0.8)	23 (6.1)
Rural	105 (83.3)	129 (100)	123 (99.2)	356 (93.9)
***5. Marital status***				
Single	5 (4)	11 (8.6)	4 (3.2)	20 (5.3)
Married	86 (68.3)	104 (80.6)	101 (81.5)	291 (76.8)
Divorced	18 (14.3)	8 (6.2)	5 (4)	31 (8.2)
Widowed	13 (10.3)	3 (2.3)	13 (10.5)	29 (7.6)
Separated	4 (3.2)	3 (2.3)	1 (0.8)	8 (2.1)
***6. Education level***				
None	13 (10.3)	41 (31.8)	20 (16.1)	74 (19.5)
Primary	82 (65.1)	67 (51.9)	80 (64.5)	229 (60.4)
Secondary	30 (23.8)	20 (15.5)	22 (17.8)	72 (19)
Tertiary	1 (0.8)	1 (0.8)	2 (1.6)	4 (1.1)
***7. Occupation***				
Business	34 (27)	19 (14.7)	29 (23.4)	82 (21.6)
Farmer	72 (57.1)	92 (71.3)	72 (58.1)	236 (62.3)
Fisher	–	2 (1.6)	7 (5.6)	9 (2.4)
Employed	6 (4.8)	–	5 (4)	11 (2.9)
Unemployed	14 (11.1)	11 (8.5)	10 (8.1)	35 (9.2)
Other	–	5 (3.9)	1 (0.8)	6 (1.6)

**Fig. 2:**
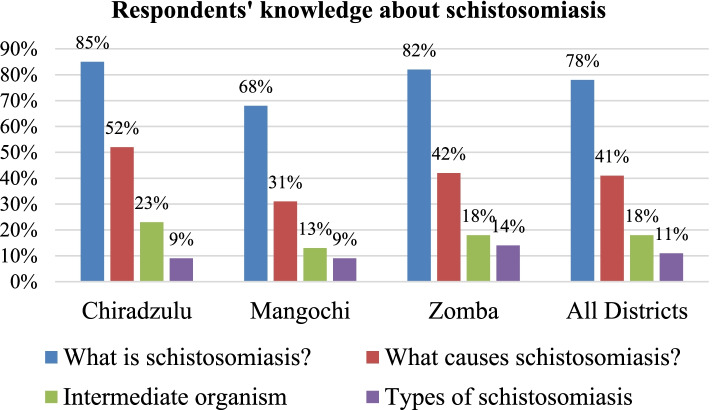
Respondents’ knowledge about schistosomiasis

Knowledge levels about schistosomiasis varied among respondents according to aspects asked about and district. The survey revealed that a majority of the respondents is highly knowledgeable about what schistosomiasis is (78%). However, respondents’ knowledge levels declined when asked to mention what causes schistosomiasis (41%), intermediate organism for schistosomiasis (18%) and the types of schistosomiasis (11%). Among the participating districts Chiradzulu generally fared better in terms of knowledge levels followed by Zomba and Mangochi (Fig. 2).

**Fig. 3:**
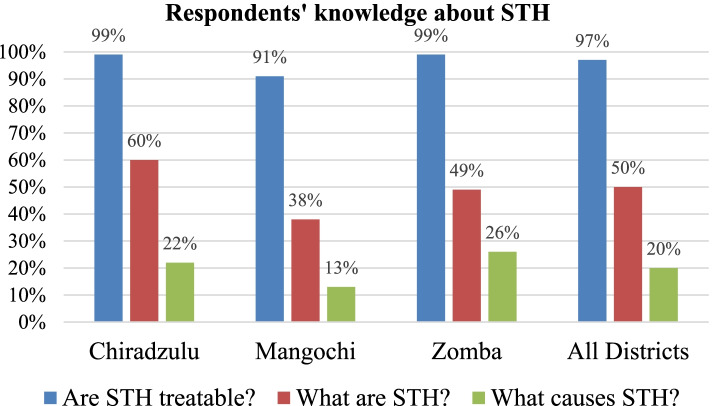
Respondents’ knowledge about STH

With regards to knowledge of STH, the survey results revealed that knowledge levels of STH varied across the study districts depending on the question that was asked (Fig. 3). Majority of the respondents were highly knowledgeable about whether STH are treatable with drugs (97%) and what STH are (50%). However, respondents had low knowledge levels when asked to mention what causes STH (20%). Among the districts, Zomba (range: 21–99%) and Chiradzulu (range: 7–99%) scored better in terms of STH knowledge levels than Mangochi (range: 9–91%).

The survey delved to inquire attitudes of respondents towards community based health service delivery process in relation to MDA. The majority expressed positive views as evidenced when 89.4% agreed that there very were few health services in the communities, which they said necessitates community members to take some of the health care responsibilities in health services delivery. They were of the view that involvement of community members in health activities will enhance health for people in their community (96.3%). As regards to community involvement in drug distribution, 93.9% opined that it helps in saving the time of the health worker to do other things, and that it is a good way to make drugs available to the people (95.2%). However, 65.2% were of the view that the distribution of drugs like praziquantel and albendazole is best done by health workers, and that communities are not capable of organizing and monitoring treatment of schistosomiasis and intestinal worms on their own (46.4%). About 68.1% of the respondents thought that community members should not handle drugs for schistosomiasis and intestinal worms because they are not trained as health workers. Only 8.4% said that community involvement in schistosomiasis and intestinal worms’ treatment is a take-over of the duties of the health worker while majority (85.5%) agreed that community members are capable of supervising the treatments of schistosomiasis and intestinal worms during MDA.

The survey sought to elucidate the issues surrounding practices related to schistosomiasis and MDA by the respondents. About a quarter (23.5%) of the respondents revealed that they had ever suffered from schistosomiasis of which 88.8% were able to receive treatment. In general about 51.4% of the respondents had recently received schistosomiasis drugs where Chiradzulu got more (58.7%) followed by Zomba (53.2%) and Mangochi (42.6%). Majority of those who received schistosomiasis drugs got them within their communities (69.7%) followed by from health centres (18.5%) and schools (11.8%). The drugs were mainly dispensed by HSA (49.7%), health centre-based health workers (45.7%) and community-based volunteers (4.6%). Asked if the respondents had experienced any problem after taking the schistosomiasis drugs, 23.6% answered affirmatively. Majority reported feelings of drowsiness or dizziness (65.2%), followed by abdominal pains (13%) and nausea or vomiting (10.9%). Less than half of the respondents (41.2%) reported that schistosomiasis drugs are readily accessible by people in villages. Table 4 summarizes practices related to schistosomiasis and MDA of respondents by districts.

**Table 4:** Practices related to schistosomiasis and MDA of respondents by districts

Practice issues asked	Number of respondents who agreed (%)			
	Chiradzulu	Mangochi	Zomba	Totals
a. Have you ever suffered from schistosomiasis?	*N* = 126 23 (18.2)	*N* = 129 36 (27.9)	*N* = 124 30 (24.2)	*N* = 379 89 (23.5)
b. If yes, did you get drugs for treatment of schistosomiasis?	*N* = 23 20 (87)	*N* = 36 34 (94.4)	*N* = 30 25 (83.3)	*N* = 89 79 (88.8)
c. Have you recently received drugs for schistosomiasis?	*N* = 126 74 (58.7)	*N* = 129 55 (42.6)	*N* = 124 66 (53.2)	*N* = 379 195 (51.4)
d. Where did you get the drugs from?	*N* = 74	*N* = 55	*N* = 66	*N* = 195
• Community	58 (78.4)	27 (49.1)	51 (77.3)	136 (69.7)
• Health facility	14 (18.9)	16 (29.1)	6 (4.1)	36 (18.5)
• School	2 (2.7)	12 (21.8)	9 (13.6)	23 (11.8)
e. Who dispensed the schistosomiasis drugs to you?	*N* = 74	*N* = 55	*N* = 66	*N* = 195
• Facility health worker	33 (44.6)	26 (47.3)	30 (45.4)	89 (45.7)
• Community health worker	36 (48.6)	27 (49.1)	34 (51.5)	97 (49.7)
• Community health volunteer	5 (6.8)	2 (3.6)	2 (3)	9 (4.6)
f. Did you experience any problem(s) after taking schistosomiasis drugs?	*N* = 74 11 (14.9)	*N* = 55 17 (30.9)	*N* = 66 18 (27.3)	*N* = 195 46 (23.6)
g. If yes, what problem did you experience after taking schistosomiasis drugs?	*N* = 11	*N* = 17	*N* = 18	*N* = 46
• Drowsiness/dizziness	4 (36.3)	11 (64.6)	15 (83.2)	30 (65.2)
• Abdominal pain	3 (27.3)	2 (11.8)	1 (5.6)	6 (13)
• Nausea/vomiting	2 (18.2)	2 (11.8)	1 (5.6)	5 (10.9)
• Other	2 (18.2)	2 (11.8)	1 (5.6)	5 (10.9)
h. Are schistosomiasis drugs readily accessible in this village?	*N* = 126 48 (38.1)	*N* = 129 61 (47.3)	*N* = 124 47 (37.9)	*N* = 379 156 (41.2)

### Assessment of MDA effectiveness

**Fig. 4:**
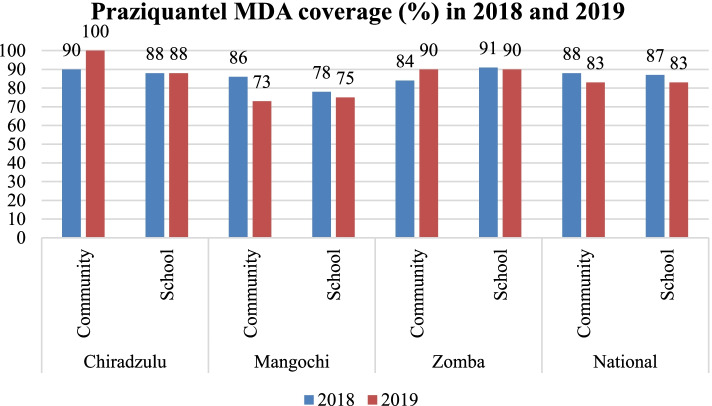
Praziquantel MDA coverage (%) in years 2018 and 2019

The study assessed MDA effectiveness targeting delivery and coverage of praziquantel and albendazole for schistosomiasis and STH respectively. MDA coverage data for these two NTD for the consecutive years of 2018 and 2019 were obtained from the three study districts. The MDA deliveries in 2018 and 2019 were done by HSA both in communities and schools in all the districts. A comparison of the coverage across the districts during the 2 years revealed that all the districts registered high coverage rates for praziquantel using community-based MDA (range: 73–100%) and using school-based MDA (range: 75–91%). For community MDA for praziquantel, Chiradzulu district scored highly above the national coverage rates in the 2 years. As for school-based MDA, Zomba and Chiradzulu districts were the highest scoring above the national rates both in 2018 and 2019. In 2019, increases in coverage rates were registered for community-based MDA in Chiradzulu and Zomba districts (Fig. 4).

**Fig. 5:**
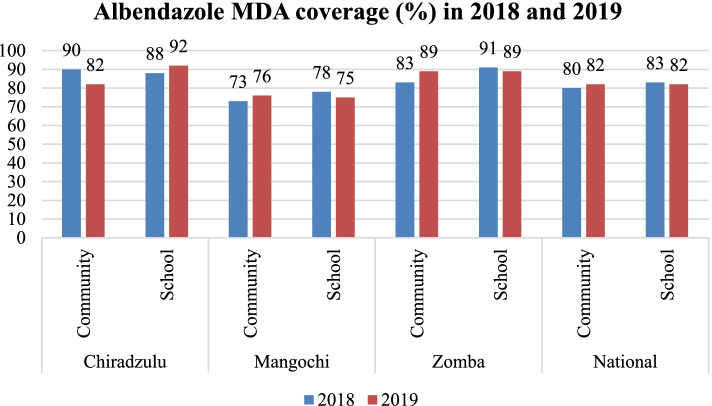
Albendazole MDA coverage (%) 2018 and 2019

Similarly, high coverage trends for community (range: 73–90%) and for schools (range: 75–92%) were observed in albendazole MDA in all the districts for 2018 and 2019. In 2018 and 2019, Chiradzulu and Zomba respectively had highest albendazole coverage using the community MDA delivery approach. As for school coverage, Zomba and Chiradzulu were highest for 2018 and 2019, respectively. In 2019, increases in coverage rates were registered for community-based MDA in Mangochi, Zomba districts and the national level; and for school-based MDA in Chiradzulu district (Fig. 5).

A comparison between albendazole MDA coverage rates for 2018 and 2019 revealed that there were no differences among the districts with regards to praziquantel and albendazole distribution. No differences were also observed for both praziquantel and albendazole between community and school modes of MDA deliveries.

### Health authorities and community perceptions on priority health issues and MDA

The study sought to understand the perceptions on priority health issues and MDA by health authorities as providers and community members as beneficiaries of health services in the three districts. On health issues, both groups agreed on Covid-19, malaria, schistosomiasis, human immunodeficiency virus and diarrhoea as health issues requiring priority attention. Some differences between perceptions of health providers and beneficiaries regarding priority health interventions were observed. The health authorities mentioned acute respiratory infections, sexually transmitted infections and tuberculosis as priority issues, while communities mentioned inadequate health workers, natural disasters, unclean water, poor sanitation, and lack of amenities such as clinics, ambulances, bicycles, protective wear, drugs, mosquito nets and health education materials as deserving priority attention. These are some of the health determinants or risk factors for the mentioned diseases.

On the community-based delivery of MDA, health authorities perceived the approach as good because it brings treatment closer to people. These sentiments were also shared by community members who exalted the role they play during MDA delivery:

*“This is a welcome idea to us communities since we are experiencing shortage of health workers in the area to deliver health services. If a community takes part in the delivery of health services, the development of the area will likely improve.”* - FGD young male, Chiradzulu

While most community members applauded delivery of MDA, some expressed misgivings because the health system prioritizes children over adults although both schistosomiasis and STH are prevalent in both groups of people in endemic areas.

*“[ … ] it is good but they give more to children ignoring the rest of us.”* - FGD adult male, Mangochi

### Availability of partners and resources for MDA

According to key informant interviews, it was learned that although MDA delivery is primarily a responsibility of the MOH through the NSCP, there are a number of partners who assist by providing various support towards implementation of MDA. The support is mostly in form of technical support, research, financial, personnel, logistics and supplies which are provided at various levels of health care system. At national level, the partnerships for MDA comprise the two Government Ministries for Health, and Education, and various non-governmental organizations who play several roles. At district level there was a presence of partners such as the German Agency for International Cooperation (GIZ) through the Nutrition and Access to Primary Education project in Chiradzulu, Save the Children in Zomba and the Blantyre Institute of Community Outreach in Mangochi who assist the districts by providing various resources such as transport and feeding pupils during MDA. However, in the study districts, the presence of partner organizations at health centre and community levels was scarce (Table 5).

**Table 5:** Implementation partnerships at national and district levels, resources and roles in MDA

No.	Name of partner	Type	Resources and role played
1	Ministry of Health	Government	Personnel, logistics and overall implementation
2	Ministry of Education	Government	Personnel and logistics
3	World Health Organization	UN^a^ agency	Technical advisory and supplies
4	Schistosomiasis Control Initiative Foundation	NGO^b^	Financial and logistics
5	World Vision International	NGO	Financial and logistics
6	The German Agency for International Cooperation	NGO	Financial and logistics
7	Save the Children	NGO	Financial and logistics
8	Centre for Health, Agriculture Development Research and Consulting (CHAD)	NGO	Financial, research and logistics
9	Blantyre Institute of Community Outreach	NGO	Financial, research and logistics
10	Research for Health Environment and Development (RHED)	NGO	Research

^a^UN United Nations

^b^NGO Non-Governmental Organization

### Community participation in MDA delivery

Although MDA delivery is inherently a community and school-based service, it is mostly the health professionals from national and district levels who are responsible for the planning and implementation. At the grassroots level, it is generally the HSA assisted by the community health volunteers and teachers who are involved in MDA delivery in communities and schools respectively. During interviews held with various health authorities and community members, the study sought to solicit their views on the question of whether it is health workers or community members who should distribute MDA. It was learned that during MDA, community involvement and participation were restricted to community sensitization and actual drug distribution. However, both health authorities and community members share positive perceptions on the use of community-based modes of delivering health services during MDA delivery. These community-based approaches enhance accessibility of the services and furthermore, allow community participation in solving their own health problem which will bring a sense of ownership and sustainability of MDA services.

*“[ … ] community participation in health delivery is good, it reduces workload for health workers”* - Medical Assistant, Chiradzulu

*“Community participation makes the work of health workers easier by supporting in some cases like emergency conditions. It helps to improve the quality of health services like immunization and to promote good hygiene practices.”* – A community leader, Zomba

### Opportunities and challenges in implementation of MDA

As the objectives of the assessment were to understand how well MDA is being implemented and to identify opportunities for improvement in MDA implementation, we analyzed data from various interviews with key informants to identify facilitators and barriers faced during implementation of MDA in the study districts. We then used the Consolidated Framework for Implementation Research to organize the identified facilitators and barriers across the eight components for MDA implementation as raised by the informants during interviews. Table 6 summarizes and reports the identified facilitators and barriers that were common across the eight MDA components, as well as those that were unique to each component.

**Table 6:** Facilitators and barriers to implementation across the eight MDA components, as commonly reported during interviews with key informants

CFIR^a^ domain	MDA^b^ component							
	Community awareness	Partnerships and collaboration	Integration with existing interventions	Training of implementers	Morbidity management	Delivery of supplies	Adequacy of implementers	Proper geographical demarcation
1). **Characteristics of MDA programme**								
*Facilitators*								
^**•**^ Control or elimination of diseases	**√**	**√**	**√**	**√**	**√**	**√**	**√**	**√**
*Barriers*								
^**•**^ Risk of developing drug resistance	**x**	**x**	**x**	**x**	**x**	**x**	**x**	**x**
2). **External environment and context**								
*Facilitators*								
• Existence of political will to control diseases	**√**	**√**				**√**	**√**	**√**
• Existence of partners at national level	**√**	**√**	**√**	**√**	**√**	**√**	**√**	**√**
• Multi-sectoral collaboration in diseases control	**√**	**√**	**√**	**√**	**√**	**√**	**√**	**√**
*Barriers*								
• Existence of research gaps/limited information	**x**	**x**	**x**	**x**	**x**		**x**	**x**
• No public-private partnerships	**x**	**x**	**x**	**x**	**x**	**x**	**x**	**x**
• Exclusion of inaccessible or hard-to-reach communities	**x**	**x**	**x**	**x**	**x**	**x**	**x**	**x**
• Community resistance due to misconceptions and beliefs	**x**	**x**	**x**	**x**	**x**	**x**	**x**	**x**
3). **Internal context and setting**								
*Facilitators*								
• Enabling policy framework	**√**	**√**	**√**	**√**	**√**	**√**	**√**	**√**
• Existence of skilled health workers at all levels of the healthcare system	**√**	**√**	**√**	**√**	**√**	**√**	**√**	**√**
• Existence of supportive community volunteers	**√**	**√**	**√**	**√**	**√**	**√**	**√**	**√**
• Capacity to deliver effective health education	**√**	**√**	**√**	**√**	**√**	**√**	**√**	**√**
*Barriers*								
• Non-existent complementary integrated interventions for diseases control	**x**	**x**	**x**	**x**	**x**	**x**	**x**	
• Lack of adequate resources for diseases control	**x**	**x**	**x**		**x**	**x**	**x**	**x**
• No linkage between NTD and WASH programmes	**x**	**x**	**x**	**x**	**x**	**x**		
• Inadequate partners at district, health centre and community levels	**x**	**x**			**x**	**x**	**x**	**x**
• Inadequate health workers in remote areas	**x**	**x**		**x**	**x**		**x**	**x**
4). **Characteristics of MDA implementers**								
*Facilitators*								
• Health education delivery skills	**√**	**√**	**√**	**√**	**√**	**√**	**√**	**√**
*Barriers*								
• Low knowledge about schistosomiasis and STH	**x**	**x**	**x**	**x**	**x**	**x**	**x**	
• Poor community engagement skills	**x**		**x**	**x**	**x**		**x**	
• Lowly motivated health workers	**x**			**x**	**x**		**x**	
5). **MDA implementation process**								
*Facilitators*								
• Convergent views on some priority health needs between health authorities and communities	**√**		**√**		**√**	**√**	**√**	
• Willingness of targeted populations	**√**	**√**	**√**		**√**	**√**	**√**	**√**
*Barriers*								
• Scheduling of MDA coincides with rainy season or major public events	**x**		**x**			**x**		
• Delayed or inadequacy of drugs and supplies	**x**	**x**	**x**		**x**	**x**		**x**
• Sidelining community in decision-making process	**x**		**x**	**x**	**x**	**x**	**x**	
• Some divergent views on priority health needs between health authorities and communities	**x**		**x**		**x**	**x**		

Source: [28]. For each MDA component where they apply, authors indicated facilitators with a checkmark (√) and barriers are indicated with a crossmark (x) from the interviews data

^a^CFIR Consolidated Framework for Implementation Research

^b^MDA Mass drug administration

## Discussion

This paper has presented an assessment of the effectiveness of MDA in prevention and control of schistosomiasis and STH in three southern Malawi districts. The findings of the assessment have revealed that the three study districts consistently achieved high MDA coverage rates in 2018 and 2019 for both schistosomiasis and STH drugs. This achievement is possible due to the collaboration through the NSCP and health care structures in the respective districts together with partners and community members. The assessment has shown that the MDA as a strategy is an effective approach for availing the much needed schistosomiasis and STH drugs to people in the involved districts. These findings agree with similar studies carried out elsewhere [36, 37]. There were no observed differences in coverage rates between MDA delivered in communities and schools; also between praziquantel and albendazole. However, despite the high coverage rates obtained in the study districts over the 2 years, there is only slow progress towards achievement of the goal to reduce burden of schistosomiasis and STH to levels of no public health importance in Malawi by 2025. Prevalence studies done in Likoma, Nkhotakota, Lilongwe, Chikwawa, including in the current study districts of Zomba and Mangochi have pointed to persistent infections and reinfections after introduction of MDA in 2009 occurring within a year despite sustained annual MDA campaigns implemented throughout the country (Table 7).

**Table 7:** List of schistosomiasis and STH prevalence estimate studies done in Malawi between 2010 and 2020

[Ref. No.]	Year of study publication	District where study was done	Sample size	Target group examined	Diagnostic technique used	Infection species targeted	Prevalence (%)^a^
[12]	2011	Mangochi	4324	Community	Filtration	*S. haematobium*	39.0
		Likoma	4131	School children	Filtration	*S. haematobium*	64.5
			130	School children	Serology	*S. haematobium*	62.3^a^
			462	Community	Filtration	*S. haematobium*	43.4
			312	Community	Kato Katz	*S. mansoni*	0.3
			312	Community	Kato Katz	*Ancylostoma* sp.	12.3
[13]	2017	Mangochi	400	School children	Filtration	*S. haematobium*	12.5
[14]	2014	Zomba	483	School age children	Filtration	*S. haematobium*	34.0
[15]	2015	Zomba	502	Community	Sedimentation	*S. haematobium*	37.8
		Lilongwe	502	Community	Kato Katz	*S. mansoni*	9.4
		Nkhotakota	516	Community	Sedimentation	*S. haematobium*	33.3
			516	Community	Kato Katz	*S. mansoni*	10.9
			576	Community	Sedimentation	*S. haematobium*	37.2
			576	Community	Kato Katz	*S. mansoni*	7.4
[16]	2013	Chikwawa	1642	Community	Sedimentation	*S. haematobium*	14.2
			1642	Community	Kato Katz	*S. mansoni*	4.3
[17]	2014	Chikwawa	165	Mothers	Filtration	*S. haematobium*	45.1
			208	Pre-school children	Filtration	*S. haematobium*	17.7
			165	Mothers	Serology	*S. haematobium*	94.5
			208	Pre-school children	Serology	*S. haematobium*	49.5
			84	Mothers	Kato Katz	*S. mansoni*	21.5
			102	Pre-school children	Kato Katz	*S. mansoni*	0
[38]	2015	Nkhotakota	143	Pre-school children	Sedimentation	*S. haematobium*	13.0
[39]	2019	Nkhotakota	242	Community	Filtration	*S. haematobium*	34.3
		Nkhotakota	315	Community	Filtration	*S. haematobium*	14.6^a^
		Lilongwe	264	Community	Filtration	*S. haematobium*	3.8
[40]	2019	Mangochi	5	Fishermen	Filtration	*S. haematobium*	80.0
[41]	2019	Mangochi	210	Fishermen	Filtration	*S. haematobium*	17.1
[42]	2020	Mangochi	520	School children	Filtration	*S. haematobium*	24.0^a^
			335	School children	Kato Katz	*S. mansoni*	8.1^a^

^a^Includes reinfections

These persistent infections and reinfections are similar to those observed in other studies carried out in Cote d’Ivoire, Kenya, Mozambique, Tanzania [43] and Togo [44]. In the wake of these revelations, there is a need to re-evaluate the current WHO guidelines that recommend that MDA delivery every 12 months in areas categorized as low-to-medium transmission settings [44–48]. Another possible factor affecting the progression towards elimination of these diseases is lack of a coordinated implementation of other interventions along with MDA for effective prevention and control of schistosomiasis and STH. These other interventions are intensified disease management to reduce morbidity, vector and ecology management for transmission control, public health services and provision of safe water, sanitation and hygiene [3, 4, 29, 36, 49, 50].

The need to engage the public in NTD prevention and control activities has become imperative in the context of morbidity reduction through MDA delivery and community participation [51]. An increase in the awareness of community members regarding schistosomiasis and STH is important for them to understand these diseases and to play a central role in their prevention and control [52]. This study has found that knowledge levels about schistosomiasis and STH varied disproportionately among survey respondents according to aspects asked about and district. This study has shown that although many respondents have heard about schistosomiasis and STH prevention and control activities, their knowledge on specific aspects of the diseases is low relative to the percentage of the respondents that know about the diseases. This disparity could be due to packaging of messages during delivery of health education for schistosomiasis and STH. The variations that were observed across the districts where Chiradzulu and Zomba fared better than Mangochi in schistosomiasis and STH knowledge levels correlate with their respective literacy levels for women/men at 77.8%/93.3% (Chiradzulu), 77.9%/87.7% (Zomba) and 57.0%/71.1% (Mangochi) [19]. These findings are similar to studies done in Nigeria, Egypt, Cameroon, Papua New Guinea, Turkey and Philippines which demonstrated that despite implementation of numerous activities towards the control of NTDs, there is little sensitization of the general public in order to increase awareness of the diseases [51–56].

The assessment has found that during MDA delivery, community involvement and participation are restricted to community sensitization and actual drug distribution times. There is a need for more involvement and participation during MDA planning and implementation in order to enhance the spirit of ownership and empowerment. Additionally, there is a need for more community participation in some aspects of delivery of the other key interventions such as case-management, vector management, environmental improvement measures and health promotion, which are recommended by WHO for prevention and control of NTD [4] in order to enhance sustainability of the control programmes. However, for the effective community participation in the prevention and control of schistosomiasis and STH there is a need for proper and better packaging of educational messages about causes and transmission of these diseases for their decision making processes. Furthermore, it was learned that there are a number of partners who assist the Government through the MOH by providing support towards implementation of various MDA related activities. Availability of partners, resources and public-private partnerships are vital in prevention and control programmes to ensure that the progress made towards schistosomiasis and STH prevention and control in Malawi is consolidated and sustained [36, 57–59]. The 2017–2022 Malawi Health Sector Strategic Plan II provides a rallying point for various health partners to participate in health delivery endeavours [20], the implementation of MDA for schistosomiasis and STH prevention and control also requires availability of partners and resources for its effective delivery [36, 57–59]. To enhance the coverage and sustainability of the MDA approach a scaling-up of the MDA delivery may benefit from experiences and use of the ExpandNet/WHO resources through “deliberate efforts to increase the impact of successfully tested health innovations so as to benefit more people and to foster policy and programme development on a lasting basis” [60]. This present study has shown that perspectives of health workers and community members on the priority needs were mostly similar, thus meeting the minimum conditions for early involvement of all stakeholders including partners and community members, in the planning of interventions. However, communities in the study districts further identified inadequate health workers, natural disasters, lack of clean water, poor sanitation, and lack of amenities such as clinics, ambulances, bicycles, protective wear, drugs, mosquito nets and health education materials as deserving priority attention. Considering the limitations in available resources, competencies as well as supportive systems in Malawi including the study districts it is more realistic for the stakeholders to explore how these expressed community needs can be addressed within the context of public-private partnerships or other means [36, 57–59]. This may also turn out to be a more sustainable approach.

Perceptions about diseases are important factors in shaping the necessary policies toward the prevention and control of such diseases. In the context of schistosomiasis and STH, perceptions and public knowledge may influence community’s decision-making for drug uptake and policy formulation for MDA implementation. In this assessment, both health authorities and community members perceived the community-based approach of MDA delivery as good because it brings treatment closer to people. However, some community members expressed a concern that the health system prioritizes children over adults although both schistosomiasis and STH are prevalent between both groups of people in endemic areas. These perceptions are similar to those expressed in other studies carried out in Turkey [55], Philippines [56], Ghana and Tanzania [61].

This paper has documented several opportunities and challenges in the implementation of MDA for prevention and control of schistosomiasis and STH. Despite the many challenges faced by the health sector in implementation of MDA, the authorities remain committed to exploit the available opportunities for improving accessibility to community health services including MDA for prevention and control of schistosomiasis and STH. These challenges are similar to those encountered in most Asian and Sub-Sahara African countries as raised in a review by Bergquist and Gray (2019) [62]. In order to tackle the challenges associated with implementation of MDA towards the elimination of NTD goal, there is a need for embracing other WHO recommended interventions and community-based health initiatives by actively engaging communities themselves.

Our study has some limitations; the data were collected from only three districts from the southern region of Malawi. Although it was not the intention from the outset that it should represent the whole country, the interviews with national authorities and reviews of official documents suggest that our findings could be generalized. The emergence of Covid-19 pandemic was a major challenge in implementation of the study such that no feedback of the findings from the qualitative interviews and focus group discussions was given to the participants for verification and consensus before report compilation raising some possibility for misrepresentation. The inclusion of highly endemic districts and use of multiple data sources are the major strengths of this study. As possible areas of future research, we would recommend inclusion of prevalence and cost-effectiveness assessments of MDA delivery to enrich the findings of similar studies, and also to test the effects of implementation of several interventions such as vector control, WASH, enviromental management, using the integrated approach in a longitudinal study in at least one of the districts with high prevalence of these NTD.

## Conclusions

Our findings indicate that implementation and effectiveness of MDA in the three target districts are satisfactory. The districts have been registering high coverage for praziquantel and albendazole both in communities and schools. However, persistent infection and reinfection rates recorded within a year after delivery of MDA are casting a doubt on the prospects of attaining the set goal of reducing the burden of schistosomiasis and STH to levels of no public health importance by 2025. This study has identified several operational challenges including lack of vital schistosomiasis and STH related knowledge by the public which are hampering efforts by the NSCP and partners towards the global NTD goals by 2030. There is an urgent need to exploit the available opportunities for promotion of community participation and partnerships as well as a coordinated implementation of other interventions along with MDA for effective prevention and control of schistosomiasis and STH.

## Supplementary Information

**Additional file 1.**

## Data Availability

The datasets generated and/or analyzed during the current study are not publicly available because participants did not grant permission for public sharing of their interview transcripts in our informed consent process, approved by Malawi National Health Sciences Research Committee but are available from the corresponding author on reasonable request.

## Abbreviations

EHP: Essential Health Package
FGD: Focus Group Discussion
GIZ: *Die Deutsche Gesellschaft für Internationale Zusammenarbeit* (The German Agency for International Cooperation)
HSA: Health Surveillance Assistant
MOH: Ministry of Health
NSCP: National Schistosomiasis and Soil-Transmitted Helminths Control Programme
MDA: Mass Drug Administration
NTD: Neglected Tropical Diseases
STH: Soil-Transmitted Helminths
WHO: World Health Organization
