# Supplementary material for: An assessment of implementation and effectiveness of mass drug administration for prevention and control of schistosomiasis and soil-transmitted helminths in selected southern Malawi districts
Source: BMC Health Serv Res. 2022 Apr 19;22:517. doi: 10.1186/s12913-022-07925-3 (PMC9016207; doi:10.1186/s12913-022-07925-3)
Supplement: Supplementary file 1 — Additional file 1. [file 12913_2022_7925_MOESM1_ESM.docx]

**DATA COLLECTION INSTRUMENTS USED IN THE STUDY**

1. **Information and procedure for eliciting informed consent**

[**Enumerator:** Please read out the project information sheet to the participant and ask for a written consent before proceeding with the survey].

Enumerator ID: /_/_/_/_/

**Project Fact Sheet**

1. **Who are the people running this project? How can I reach them?**

| **No** | **Name** | **Position** | **Organization** | **Contact** | **Telephone(s)** |
| --- | --- | --- | --- | --- | --- |
|  |  |  |  |  |  |
|  |  |  |  |  |  |
|  |  |  |  |  |  |
|  |  |  |  |  |  |
|  |  |  |  |  |  |
|  |  |  |  |  |  |

**What is the purpose of this project?**

The purpose of this study is to collect information on the factors that influence community participation and the experiences of community members, community service providers and direct beneficiaries of disease interventions. We are interested in those interventions that are provided by the community. In particular, this is to increase community ownership of MDA for control of schistosomiasis and STH in rural Malawi districts. Through interviews and observations, this study will explore and allow us to better understand the extent to which community can participate in the intervention against schistosomiasis and intestinal worms that affect them.

**What area of health is the project interested?**

Project staff is interested in how the community in this area, with the facilitation of the formal health service/system, will participate in the intervention/s against neglected tropical diseases as identified by the communities such as schistosomiasis and intestinal worms.

**How will project staff gather information?**

Project staff will gather information through conducting interviews, discussions and by observations in the study districts.

**How will the findings from this project be used?**

Findings may be published in professional journal articles, exhibits, books, reports and used in educational manuals. Recommendations derived from this study will be useful for global health including health systems researchers in developing countries initiating national and local programmes for the management of neglected tropical diseases with the direct participation of affected communities.

**What is important to know for communities/individuals participating in the project?**

All data collected through the in-depth interviews, discussions, survey questionnaires, and during observation sessions will be kept confidential. Communities and individuals will be asked to sign informed consent to assure that they understand the purpose of the project, what the project is about, and that they can choose to withdraw participation at any time.

**Informed consent - Interviews (individuals and community)**

There are many organizations that would like to have the communities participating in interventions against diseases that affect them so as to reduce the time and cost of intervention. The Ministry of Health through the NTD programme and its partners are collecting information on people’s experiences while directing intervention at the community level. We will ask questions about many aspects of life in this community (household/family). We want to learn more about your experience with community based interventions. We may take pictures of activities in the community, which will help us to explain our findings. We will go to different households, talk with women and men in this community (household) either in groups or individually. Some of the conversation may be recorded so that we do not miss out some of the important things that are said. If you choose to be in this study, we will ask you questions about your personal experiences. The questions are general but if you find that some questions are not going well with you, please do not feel compelled to answer any of them for any reason. We will talk to you for about **30-60 minutes**. You can decide if you want to take part or opting out in this study. Taking part in this study will not cost you anything. You may also leave the study at any time. You can leave for any reason without any problems. You and your family may not get any direct benefits from being in the study. However, what you tell us will help us developing a strategy for training community members to treat schistosomiasis and intestinal worms within the community and thus improve the health of the people in this community. Your name and what you say to us for this study will be kept private as much as the law allows.

Do you have any questions about the study? If you have any questions about your rights in the study or any other questions, you may contact XXXX during the study and in the future. If you have concerns about human rights, ethics and welfare issues you may contact the National Health Sciences Research Committee (NHSRC) of the Ministry of Health at XXXX.

If you agree to answer our questions, you can tell us that you agree by repeating these words and then putting your name and signature in the space below.

***I have read the foregoing information, or it has been read to me. I have had the opportunity to ask questions about it and any questions I have been asked have been answered to my satisfaction. I consent voluntarily to participate in this study and understand that I have the right to withdraw from the study at any time without in any way affecting my further medical care.***

__________________________________________________ _______________

Community Leader/Individual Participant’s name/signature Date

_________________________________________________ _______________

Interpreter/Witness’s Signature Date

1. **A Structured Knowledge Attitudes and Practices (KAP) Survey Questionnaire at Household Level**

**Introduction:** The SCHIMDA Project, would like to request for your kind cooperation to participate in this survey in order to collect information from you to assess the level of knowledge, attitudes and practices related to schistosomiasis and soil transmitted helminths (STH) or intestinal worms in this community.

1. Have you read out the project information sheet and a written informed consent has been granted by the respondent or his/her parent/guardian? **1** = Yes (Proceed with the interview)

**Instructions:** Please indicate the responses by choosing the corresponding codes. Where the options are **correct** or **incorrect**, please refer to the guiding notes at the end of this questionnaire.

**Part A: Geographical information**

1. Districts:

**1** = Chiradzulu; **2** = Mangochi; **3** = Zomba

1. Health Centres:

**11** = Maravi, **12** = Namadzi, **13** = Namitambo, **14** = Nkalo, **21** = Chilonga; **22** = Makanjira; **23** = Malombe; **24** = Mtimabii, **31** = Chingale, **32** = Matiya, **33** = Mayaka, **34** Namadidi.

1. Villages:

**11 Maravi Health Centre**

**111** = Kalanje

**112** = Likoswe

**113** = Mkwate

**114** = Ngusiche

**12 Namadzi Health Centre**

**121** = Luna

**122** = Malukula

**123** = Masuku

**124** = Mitawa

**13 Namitambo Health Centre**

**131** = Khumbunya

**132** = Likovo

**133** = Muhasuwa

**134** = Nnamala

**14 Nkalo Health Centre**

**141** = Makalani

**142** = Masuso

**143** = Nkhupela

**144** = Tambala

**21 Chilonga Health Centre**

**211** = Chilonga

**212** =Kela

**213** = Makunula

**214** = Maloya

**22 Makanjira Health Centre**

**221** = Chilawe

**222** = Malamia

**223** = Mikochi

**224** = Mpangama

**23 Malombe Health Centre**

**231** = Kadewere

**232** = Mkata

**233** = Nalikolo

**234** = Mkata

**24 Mtimabii Health Centre**

**241** = Lisewa

**242** = Maoni

**243** = Mwatakata

**244** = Mwenyemusa

**31 Chingale Health Centre**

**311** = Mdalakamuyanja

**312** = Mlusu

**313** = Namwiyo

**314** = Ntiku

**32 Matiya Health Centre**

**321** = Chaima

**322** = Chinkhwangwa

**323** = Mwaliwa

**324** = Naphome

**33 Mayaka Health Centre**

**331** = Amiteche

**332** = Harry

**333** = Mukhwayi

**334** = Tambala

**34 Namadidi Health Centre**

**341** = Issa

**342** = Maselema

**343** = Mbulukuta

**344** = Napwanga

1. Location: **1** = Rural; **2** = Urban
2. Ecological: **1** = Lowland; **2** = Highland

**Part B: Socio-demographic information for respondent**

1. Respondent ID number: **/-/-/-/**
2. Age: **/-/-/** = (**##** years; **00** = Do not know or remember)
3. Sex: **1** = Male; **2** = Female
4. Marital status: **1** = Single; **2** = Married; **3** = Divorced; **4** = Widowed; **5** = Separated; **6** = Other
5. Number of people in the household: Total = **/-/-/**; [Male = **/-/-/**; Female = **/-/-/**;] [Adults (> 18 years) = /-/-/; Young (< 18 years) = /-/-/]
6. Tribe: **1** = Chewa; **2** = Lomwe; **3** = Mang’anja; **4** = Ngonde; **5** = Ngoni; **6** = Sena; **7** = Tonga; **8** = Tumbuka; **9** = Yao; **0** = Other
7. Religion: **1** = Atheist; **2** = Christian; **3** = Moslem; **4** = Traditional; **5** = Other
8. Highest education level: **1** = Primary; **2** = Secondary; **3** = Tertiary; **4** = None
9. Occupation: **1** = Business; 2 = Farmer; **3** = Fisher; **4** = Employed; **5** = Schooling; **6** = Unemployed; **7** = Other

**Part C: Knowledge and practices about schistosomiasis and intestinal worms**

1. Do you know what schistosomiasis is? **1** = Yes; **2** = No
2. If yes, please tell me what schistosomiasis is? **1** = Correct; **2** = Incorrect
3. Do you know what causes schistosomiasis? **1** = Yes; **2** = No
4. If yes, what is it that causes schistosomiasis? **1** = Correct; 2 = Incorrect
5. How many types of schistosomiasis do you know?

**1** = One type; **2** = Two types; **3** = More types; **4** = Do not know

1. Do you know the signs or symptoms of schistosomiasis? **1** = Yes; **2** = No
2. If yes, please mention any sign or symptoms of schistosomiasis you know? **1** = Correct; **2** = Incorrect
3. Which organs in the body does the schistosomiasis parasites attack? **1** = Bladder only; **2** = Abdomen only; 3 = Bladder and abdomen; **4** = Other organs; **5** = Do not know
4. Do you know how healthy people get infected with schistosomiasis? **1** = Yes; **2** = No
5. If yes, explain to me how a healthy person can get infected with schistosomiasis? **1** = Correct; **2** = Incorrect
6. Do you know how schistosomiasis is transmitted from a sick person to a healthy person? **1** = Yes; **2** = No
7. If yes, explain to me how schistosomiasis is transmitted from a sick person to a healthy person? **1** = Correct; **2** = Incorrect
8. Do you know what organism acts as intermediate host for schistosomiasis parasites? **1** = Yes; **2** = No, **3** = Do not know
9. If yes, what organism acts as an intermediate host for schistosomiasis parasites? **1** = Snails; **2** = Others; **3** = Do not know
10. Do you know how one can prevent contracting schistosomiasis? **1** = Yes; **2** = No
11. If yes, how can one prevent contracting schistosomiasis? **1** = Correct; **2** = Incorrect
12. Is schistosomiasis treatable by drugs? **1** = Yes; **2** = No
13. Do you know what problem(s) schistosomiasis can cause to a person if not treated? **1** = Yes; **2** = No
14. If yes, mention any example of problems that schistosomiasis can cause to a person if not treated? **1** = Correct; **2** = Incorrect
15. Have you ever suffered from schistosomiasis? **1** = Yes; **2** = No
16. If yes, did you get drugs for treatment of schistosomiasis? **1** = Yes; **2** = No
17. Have you recently received drugs for schistosomiasis? **1** = Yes; **2** = No
18. If yes, when did you recently receive the drugs for schistosomiasis?

**1** = Within past one week; **2** = Within a month ago; **3** = Within one year ago; **4** = Over one year ago; **5** = Do not remember

1. Where did you get the drugs from?

**1** = Health Facility; **2** = In Community; **3** = Store or grocery; **4** = School; **5** = Other

1. Who dispensed the schistosomiasis drugs to you?

**1** = Facility health worker; **2** = Community health worker; **3** = Community volunteer; **4** = Other

1. Did you experience any problem(s) after taking schistosomiasis drugs? **1** = Yes; **2** = No
2. If yes, what problem did you experience after taking schistosomiasis drugs?

**1** = Drowsiness/dizziness; **2** = Nausea/vomiting; **3** = Abdominal pain; **4** = Fever/sweating **5** = Allergy/rash; **6** = Others

1. How many other members of this household received drugs for schistosomiasis within the past year? **/-/-/**
2. Are schistosomiasis drugs readily accessible in this village? **1** = Yes; **2** = No
3. Do you know what intestinal worms are? **1** = Yes; **2** = No
4. If yes, please tell me what intestinal worms are? **1** = Correct; **2** = Incorrect
5. Do you know what causes intestinal worms? **1** = Yes; **2** = No
6. If yes, what causes intestinal worms? **1** = Correct; 2 = Incorrect
7. Do you know how healthy people get infected with intestinal worms? **1** = Yes; **2** = No
8. If yes, explain to me how a healthy person can get infected with intestinal worms? **1** = Correct; **2** = Incorrect
9. Do you know how intestinal worms are transmitted from a sick person to a healthy person? **1** = Yes; **2** = No
10. If yes, explain to me how intestinal worms are transmitted from a sick person to a healthy person? **1** = Correct; **2** = Incorrect
11. Do you know how one can prevent contracting intestinal worms? **1** = Yes; **2** = No
12. If yes, how can one prevent contracting intestinal worms? **1** = Correct; **2** = Incorrect
13. Are intestinal worms treatable by drugs? **1** = Yes; **2** = No
14. Do you know what problem(s) intestinal worms can cause to a person if not treated? **1** = Yes; **2** = No
15. If yes, mention any example of problems that intestinal worms can cause to a person if not treated? **1** = Correct; **2** = Incorrect
16. Have you ever suffered from intestinal worms? **1** = Yes; **2** = No
17. If yes, did you get drugs for treatment of intestinal worms? **1** = Yes; **2** = No
18. Have you recently received drugs for intestinal worms? **1** = Yes; **2** = No
19. If yes, when did you recently receive the drugs for intestinal worms?

**1** = Within past one week; **2** = Within a month ago; **3** = Within one year ago; **4** = Over one year ago; **5** = Do not remember

1. Where did you get the drugs for intestinal worms from?

**1** = Health Facility; **2** = In Community; **3** = Store or grocery; **4** = School; **5** = Other

1. Who dispensed the intestinal worms’ drugs to you?

**1** = Facility health worker; **2** = Community health worker; **3** = Community volunteer; **4** = Other

1. Did you experience any problem(s) after taking intestinal worms drugs? **1** = Yes; **2** = No
2. If yes, what problem did you experience after taking drugs for intestinal worms?

**1** = Drowsiness/dizziness; **2** = Nausea/vomiting; **3** = Abdominal pain; **4** = Fever/sweating **5** = Allergy/rash; **6** = Others

**Part D: Respondents’ attitude towards health services delivery in community**

Some people often make some statements about the community based service delivery process. Kindly indicate your agreement or disagreement with the following statements by ticking in the appropriate corresponding box.

| No. | Statements | **1** = Strongly  agree | **2** = Agree | **3** = Indifference | **4** = Disagree | **5** = Strongly  disagree |
| --- | --- | --- | --- | --- | --- | --- |
|  | Because there are few health services in the communities, community members have to take some health care responsibilities. |  |  |  |  |  |
|  | Distribution of drugs like Praziquantel and Albendazole is best done by health workers. |  |  |  |  |  |
|  | Communities are not capable of organizing and monitoring treatment of schistosomiasis and intestinal worms on their own. |  |  |  |  |  |
|  | The involvement of community members in health activities is possible with frequent monitoring and supervision by health staff. |  |  |  |  |  |
|  | Community members should not handle drugs for schistosomiasis and intestinal worms because they are not trained health workers. |  |  |  |  |  |
|  | The involvement of community members in health and development activities will enhance health for people in this community. |  |  |  |  |  |
|  | Community members are quite capable of supervising the treatments schistosomiasis and intestinal worms during the continuation phase. |  |  |  |  |  |
|  | Community involvement in drug distribution saves the time of the health worker to do other things. |  |  |  |  |  |
|  | Community based delivery of drugs is a good way to make drugs available to the people. |  |  |  |  |  |
|  | Community involvement in schistosomiasis and intestinal worms’ treatment is a take-over of the duties of the health worker. |  |  |  |  |  |

**END OF INTERVIEW**

**Please thank the respondent for sparing time to answer the questions**

**GUIDING NOTES**

1. Schistosomiasis is a disease caused by small worms which infect a person when he gets into contact with infested water, causing bloody urine or stool, abdominal pain, fever and other symptoms
2. Schistosomiasis is caused by small worms which are spread by snails, which are living in freshwaters of rivers, dams and lakes
3. Signs or symptoms of schistosomiasis:
4. Abdominal pain
5. Fever
6. Blood in urine
7. Blood in stool / faeces
8. Painful urination
9. Cough
10. Headache
11. Body aches
12. Rash
13. Abdominal swelling (ascites)
14. A healthy person can get infected with schistosomiasis by getting into contact with infested waters with small worms from particular snails in rivers, dams and lakes during routine household chores (washing, bathing, drawing water for home use), recreational (swimming, playing) and income generating activities (fishing, farming, business travel).
15. Schistosomiasis is transmitted from a sick person to healthy person by the former urinating or defecating into waters having particular snails, thereby infecting the snails and after some weeks, the snails release small worms (cercariae) which then penetrate the latter while in the same waters.
16. One can prevent contracting schistosomiasis by avoiding contact with infested water.
17. Problems that schistosomiasis can cause to a person if not treated include:
18. Abdominal swelling (ascites)
19. Lesions in genital areas
20. Paralysis
21. Seizures
22. Intestinal worms are diseases which are caused by worms which reside and affect intestines which commonly cause children.
23. Intestinal worms’ diseases are caused by roundworms, pinworms, hookworms
24. Healthy person can get infected with intestinal worms though eating food contaminated with eggs of worms or hookworms penetrating bare feet of those in contact with infected stool / faeces
25. Intestinal worms are transmitted from a sick person to healthy person through poor disposal of human waste (stool / faeces) by the former which gets in contact with the latter
26. One can prevent contracting intestinal worms by practising good hygiene practices (handwashing, proper disposal of human waste), good food preparations, putting footwear
27. Problems that intestinal worms can cause to a person if not treated include:
28. Abdominal pains
29. Diarrhoea, nausea, vomiting
30. Fatigue / tiredness
31. Unexplained weight loss
32. Anaemia
33. Stunting / malnutrition
34. Poor cognitive function resulting in worsening school performance
35. Bowel obstruction or rectal prolapse
36. **Semi-structured Questionnaire for Health Workers (Facility and Community based)**

**Instructions:**

1. **Who to interview:** Health workers. These include:

- Clinician in-charge
- Nurse
- Assistant Environmental Health Officer
- Senior Health Surveillance Assistant
- Health surveillance Assistant

1. **Sample:** Select **one** health worker from each study health centre making it **four** health workers (two male and two female) from each study district. This brings to **twelve** the number of health workers to be interviewed in all three study districts.
2. **When:** Administer instrument before and after project intervention

**District Code: [__] Health Centre Code: [__][__] Village Code: [__][__][__]Enumerator: ______________ Date: __/__/__**

The Ministry of Health through the Schistosomiasis and Intestinal worms Mass Drug Administration (SCHIMDA) Project intends to use the Community for delivery of health interventions against Schistosomiasis and intestinal worms. In this context, the Community, the Health Services and Other Partners have specific roles to perform in order to ensure the implementation of these interventions.

Please would you like to take part and provide information to the following questions? Information provided will be kept strictly confidential. [If yes, proceed with interview. If no, Stop].

**Position in the Health worker:** ______________________ **Name (Optional): _____________**

**Number of years as Health worker:** _____________ **Sex:** 1=Male [__] 2=Female [__] **Highest education level attained:** _______________________

1. What are some of the health services being provided at your health centre?
2. What are some of health services being implemented in the communities or villages?
3. How are these services provided to the people? i.e. facility based, outreach, health worker driven or community based/volunteers? What determines the mode of delivery? Explain.
4. What is your perception of community participation in delivery of health services at health centre and community levels? Please explain your answer.
5. What is your perception of community contribution towards delivery of health services at health centre and community levels?
6. In your understanding, what are the priority health needs and issues in the villages in the catchment area of your health facility?
7. Apart from government, who are other partners involved in delivery of health services at this health centre and in the communities? What are they doing? How are they doing it? Target groups? Target areas? Probe on community involvement.
8. What is your perception of use of incentives during delivery of health services at community level?
9. What are the likely consequences of giving gifts (such as money, materials like bicycles, food items, farm crops) to community people who are involved in the management of diseases in the community? Please explain your answer.
10. Are community members always available to provide support to the health workers in their communities?
11. If no to the above question, how can such a situation be improved?
12. What challenges do the health workers experience in meeting up with the demands of delivering health services in the communities? Please explain your answer.
13. How can these difficulties be overcome?
14. Some people often make some statements about the community based service delivery process. Kindly indicate your agreement or disagreement with the following statements by giving your opinion.

|  | **Statements** | 1 = Strongly  agree | 2 = Agree | 3 = Indifference | 4 = Disagree | 5 = Strongly  disagree |
| --- | --- | --- | --- | --- | --- | --- |
|  | Because there are few health services in the communities, community members have to take some health care responsibilities. |  |  |  |  |  |
|  | Distribution of drugs like Praziquantel and Albendazole is best done by health workers. |  |  |  |  |  |
|  | Communities are not capable of organizing and monitoring treatment of schistosomiasis and intestinal worms on their own. |  |  |  |  |  |
|  | The involvement of community members in health activities is possible with frequent monitoring and supervision by health staff. |  |  |  |  |  |
|  | Community members should not handle drugs for schistosomiasis and intestinal worms because they are not trained health workers. |  |  |  |  |  |
|  | The involvement of community members in health and development activities will enhance health services in this community. |  |  |  |  |  |
|  | The involvement of community members in health and development activities will enhance health for people in this community. |  |  |  |  |  |
|  | Community members are quite capable of supervising the treatments schistosomiasis and intestinal worms during the continuation phase. |  |  |  |  |  |
|  | Community involvement in drug distribution saves the time of the health worker to do other things. |  |  |  |  |  |
|  | Community based delivery of drugs is a good way to make drugs available to the people. |  |  |  |  |  |
|  | Community involvement in schistosomiasis and intestinal worms’ treatment is a take-over of the duties of the health worker. |  |  |  |  |  |

1. **Semi-structured Questionnaire for Community Leaders**

**Instructions:**

1. **Who to interview:** Community leaders. These include:

- Traditional leader
- Women leader
- Community development group leader
- Major CBO leader
- Religious group leaders

1. **Sample:** Select **two** community leaders, one female and one male from any two groupings in any randomly selected two villages of each health centre. This brings to **eight** the number of community leaders to be interviewed in each study district.
2. **When:** Administer instrument before and after project intervention

**District Code: [__] Health Centre Code: [__][__] Village Code: [__] [__][__] Enumerator: ______________ Date: __/__/__**

The Ministry of Health through the Schistosomiasis and Intestinal worms Mass Drug Administration (SCHIMDA) Project intends to use the Community for delivery of health interventions against Schistosomiasis and intestinal worms. In this context, the Community, the Health Services and Other Partners have specific roles to perform in order to ensure the implementation of these interventions.

Please would you like to take part and provide information to the following questions? Information provided will be kept strictly confidential. [If yes, proceed with interview. If no, Stop].

**Position in the Community:** ______________________ **Education: _____________**

**Number of years as Community leader:** _____________ **Occupation: ________________**

**Sex:** 1=Male [__] 2=Female [__]

1. What are some of the health services being provided at your nearest health centre?
2. What are some of the health services being implemented at community level?
3. How are these services provided to the people? i.e. facility based, outreach, health worker driven or community based/volunteers?
4. What is your perception of community participation in delivery of health services at health centre and community levels? Please explain your answer.
5. What is your perception of community contribution towards delivery of health services at health centre and community levels?
6. In your understanding, what are the priority health needs and issues in the villages in the catchment area of your health facility?
7. Apart from government, who are other partners involved in delivery of health services at this health centre and in the communities? What are they doing? How are they doing it? Target groups? Target areas? Probe on community involvement.
8. What is your perception of use of incentives during delivery of health services at community level?
9. What are the likely consequences of giving gifts (such as money, materials like bicycles, food items, farm crops) to community members/volunteers who are involved in the management of diseases in the community? Please explain your answer.
10. Are community members always available to provide support to the health workers in their communities? Please explain. What are the enabling factors? What are the challenges?
11. If no to the above question, how can such a situation be improved?
12. What challenges do the health workers experience in meeting up with the demands of delivering health services in the communities? Please explain your answer.
13. How can these difficulties be overcome?
14. Some people often make some statements about the community based service delivery process. Kindly indicate your agreement or disagreement with the following statements by giving your opinion.

|  | **Statements** | **1** = Strongly  agree | **2** = Agree | **3** = Indifference | **4** = Disagree | **5** = Strongly  disagree |
| --- | --- | --- | --- | --- | --- | --- |
|  | Because there are few health services in the communities, community members have to take some health care responsibilities. |  |  |  |  |  |
|  | Distribution of drugs like Praziquantel and Albendazole is best done by health workers. |  |  |  |  |  |
|  | Communities are not capable of organizing and monitoring treatment of schistosomiasis and intestinal worms on their own. |  |  |  |  |  |
|  | The involvement of community members in health activities is possible with frequent monitoring and supervision by health staff. |  |  |  |  |  |
|  | Community members should not handle drugs for schistosomiasis and intestinal worms because they are not trained health workers. |  |  |  |  |  |
|  | The involvement of community members in health and development activities will enhance health services in this community. |  |  |  |  |  |
|  | The involvement of community members in health and development activities will enhance health for people in this community. |  |  |  |  |  |
|  | Community members are quite capable of supervising the treatments schistosomiasis and intestinal worms during the continuation phase. |  |  |  |  |  |
|  | Community involvement in drug distribution saves the time of the health worker to do other things. |  |  |  |  |  |
|  | Community based delivery of drugs is a good way to make drugs available to the people. |  |  |  |  |  |
|  | Community involvement in schistosomiasis and intestinal worms’ treatment is a take-over of the duties of the health worker. |  |  |  |  |  |

1. **Checklist for Interview and Observation of Critical Factors for Delivery of Praziquantel and Albendazole for Treatment of Schistosomiasis and Intestinal Worms at District and Health Facility Levels**

**Instructions:**

1. **Who to interview:** Programme Coordinators for Schistosomiasis and STH in each of the study districts and Health Facility In-charges
2. **Sample:** Select **one** Programme Coordinator for each district and **one** officer in-charge for every Health Centre.
3. **When:** Administer instrument before and after intervention

**District Code: [__] Health Centre Code: [__][__]Enumerator: ______________ Date: __/__/__**

The Ministry of Health through the Schistosomiasis and Intestinal worms Mass Drug Administration (SCHIMDA) Project intends to use the Community for delivery of health interventions against Schistosomiasis and intestinal worms. In this context, the Community, the Health Services and Other Partners have specific roles to perform in order to ensure the implementation of these interventions.

Please would you like to take part and provide information to the following questions? Information provided will be kept strictly confidential. [If yes, proceed with interview. If no, Stop].

**1. Existence of supporting policy. (Tick as appropriate)**

|  | By Interview | | By Observation | | Comments |
| --- | --- | --- | --- | --- | --- |
|  | Yes | No | Yes | No |  |
| Is there a written policy to support to support MDA for |  |  |  |  |  |
| Treatment of schistosomiasis |  |  |  |  |  |
| Treatment of intestinal worms |  |  |  |  |  |
| Implementation the stated policies or guidelines |  |  |  |  |  |
| Opportunities |  |  |  |  |  |
| Challenges |  |  |  |  |  |

**2. Availability of drugs or products.**

| State no. of designated centres for which drugs or products are available? | No. Available | No. not available | Comments |
| --- | --- | --- | --- |
| Treatment of schistosomiasis |  |  |  |
| Treatment of intestinal worms |  |  |  |
| Stock outs of these drugs |  |  |  |

**3. Availability of transport**

| Is there functioning means of transport for MDA and supervision? | By Interview | | By Observation | | Comments |
| --- | --- | --- | --- | --- | --- |
|  | Yes | No | Yes | No |  |
|  |  |  |  | |  |
| How do you cope in the face of challenges, if any? |  |  |  | |  |

**4. Budget for transport for supervision**

| Is there a current budget for transportation for supervision of: | By Interview | | By Observation | | Comments |
| --- | --- | --- | --- | --- | --- |
|  | Yes | No | Yes | No |  |
| Treatment of schistosomiasis |  |  |  |  |  |
| Treatment of intestinal worms |  |  |  |  |  |

**5. Availability of supervision team**

| Is there a supervision team at district and health centre levels for: | By Interview | | By Observation | | Comments |
| --- | --- | --- | --- | --- | --- |
|  | Yes | No | Yes | No |  |
| Treatment of schistosomiasis |  |  |  |  |  |
| Treatment of intestinal worms |  |  |  |  |  |

**6. Availability of storage space**

| How many of your health facilities have space for storage of commodities for: | By Interview | | By Observation | | Comments |
| --- | --- | --- | --- | --- | --- |
|  | No. with space | No. without space | No. with space | No. without space |  |
| Treatment of schistosomiasis |  |  |  |  |  |
| Treatment of intestinal worms |  |  |  |  |  |

**7. Incorporation of MDA in District Implementation Plans we can ask why and why not?**

| Does the 2020 district health plan incorporate MDA for: | By Interview | | By Observation | | Comments |
| --- | --- | --- | --- | --- | --- |
|  | Yes | No | Yes | No |  |
| Treatment of schistosomiasis |  |  |  |  |  |
| Treatment of intestinal worms |  |  |  |  |  |
| For response above, explain why or why not? |  |  |  |  |  |

**8. Availability of budget for MDA**

| Is there a budget for MDA in 2020 for: | By Interview | | By Observation | | Comments |
| --- | --- | --- | --- | --- | --- |
|  | Yes | No | Yes | No |  |
| Treatment of schistosomiasis |  |  |  |  |  |
| Treatment of intestinal worms |  |  |  |  |  |

**9. Availability of a register on stock management**

| Is there a register on stock management for: | By Interview | | By Observation | | Comments |
| --- | --- | --- | --- | --- | --- |
|  | Yes | No | Yes | No |  |
| Treatment of schistosomiasis |  |  |  |  |  |
| Treatment of intestinal worms |  |  |  |  |  |

**10. Reporting for MDA**

| For how many facilities in year 2020 did you receive report/returns for: | <50% | 50-75% | >75% | <50% | 50-75% | >75% | Comments |
| --- | --- | --- | --- | --- | --- | --- | --- |
| Treatment of schistosomiasis |  |  |  |  |  |  |  |
| Treatment of intestinal worms |  |  |  |  |  |  |  |

**11. Any challenges and recommendations?**

__________________________________________________________________________________________________________________________________________________________________________________________________________________________________________

1. **In-depth Interview Guide for Partners**

**Instructions:**

1. **Who to interview:** All focal persons for NGOs, Donor agencies, MOH and UN agencies operating with the study Districts as well as CBOs and community groups.
2. **Sample:** Select **one** focal person for each group (NGO, Donor agencies, MOH and UN agencies) in the study Districts as well as the CBOs and community groups.
3. **When:** Administer instrument before and after intervention

**District: ________________ [__] Type of organization: NGO [__] CBO [__] Community Group [__] Position of interviewee: _______________________________ Date: __/__/__**

**Introduction:**

Good day. I am ……………… … I am from Ministry of Health and wish to learn about how you feel about participating in the delivery of Mass Drug Administration (MDA) for schistosomiasis and intestinal worms treatment. We will ask you questions about your personal experience. The questions are general but if you find that some questions are not well with you, please do not feel compelled to answer any of them for any reason. We will talk to you for about 10 - 15 minutes. Participation in this interview is voluntary and you may choose to terminate the interview if you so decide without any repercussion. What you tell us will help us develop a strategy for training community members to manage the interventions stated earlier and thus improve the health of the people. Do you have any questions about the study? If you have any questions about your rights in the study or in case of emergency, you may contact XXXX or XXXXX or the National Health Sciences Research Committee on +265 XXXX. Are you willing to participate? Yes [__] No [__]

**If No, Thank respondent and terminate interview.**

**A: Perception of partners towards MDA**

1. How did you get involved in the MDA activities and when?
2. Why did your organization decide to get involved in MDA activities?
3. What role does your organization play in the partnership?
4. What challenges does your organization experience in the partnerships? Please explain.

**B: Special arrangements for dialogue, decision-making and problem solving.**

1. What arrangements has your organization put for dialogue and problem solving within the partnership?
2. How do they function?
3. How have these arrangements influenced the MDA? Probe for positive and negative impacts.

**Section C: Assessing the MDA intervention**

1. What are the benefits of the MDA intervention? (Probe for economic and social benefits)
2. What are the costs implications for MDA intervention? (Probe for economic, social and opportunity costs)
3. What can you say about the complexity levels of the intervention? Which ones were easier for the community to implement and which one were difficult?

**Name of interviewer:** ____________________

1. **Checklist for Interview and Observation of Critical Factors for MDA of Praziquantel and Albendazole for treatment of Schistosomiasis and Intestinal Worms (Partnership Structures e.g. NGOs, Donors, etc.)**

**Instructions:**

1. **Who to interview:** All focal persons for NGOs, Donor agencies, MOH and UN agencies operating with the study Districts.
2. **Sample:** Select 1 focal person for each group (NGO, Donor agencies, MOH and UN agencies) in the study Districts.
3. **When:** Administer instrument before and after intervention

**District Code: [__] Health Centre Code: [__][__] Village Code: [__] [__][__] Identification Code: [__][__][__] Enumerator: ______________ Date: __/__/__**

The Ministry of Health through the Schistosomiasis and Intestinal worms Mass Drug Administration (SCHIMDA) Project intends to use the Community for delivery of health interventions against Schistosomiasis and intestinal worms. In this context, the Community, the Health Services and Other Partners have specific roles to perform in order to ensure the implementation of these interventions.

Please would you like to take part and provide information to the following questions? Information provided will be kept strictly confidential. [If yes, proceed with interview. If no, Stop].

Fill in the appropriate boxes

|  | By Interview | | By Observation | Comments |
| --- | --- | --- | --- | --- |
|  | NGOs | Donors | Local partners |  |
| 1. How many partners are involved in MDA at district level? |  |  |  |  |
| - Treatment of schistosomiasis |  |  |  |  |
| - Treatment of intestinal worms |  |  |  |  |
| 2. How many are involved in routine planning and budgeting for? |  |  |  |  |
| - Treatment of schistosomiasis |  |  |  |  |
| - Treatment of intestinal worms |  |  |  |  |
| 3. How many partners provide direct funding for MDA for? |  |  |  |  |
| - Treatment of schistosomiasis |  |  |  |  |
| - Treatment of intestinal worms |  |  |  |  |
| 4. How many provide logistic support (transport, commodities) for MDA for? |  |  |  |  |
| - Treatment of schistosomiasis |  |  |  |  |
| - Treatment of intestinal worms |  |  |  |  |
| 5. How many provide training for MDA for? |  |  |  |  |
| - Treatment of schistosomiasis |  |  |  |  |
| - Treatment of intestinal worms |  |  |  |  |
| 6. How many provide support for advocacy for MDA for? |  |  |  |  |
| - Treatment of schistosomiasis |  |  |  |  |
| - Treatment of intestinal worms |  |  |  |  |
| 7. What are the challenges of MDA for: |  |  |  |  |
| - Treatment of schistosomiasis |  |  |  |  |
| - Treatment of intestinal worms |  |  |  |  |
| 8. What are opportunities offered by MDA for: |  |  |  |  |
| - Treatment of schistosomiasis |  |  |  |  |
| - Treatment of intestinal worms |  |  |  |  |
| 9. What recommendations of MDA for: |  |  |  |  |
| - Treatment of schistosomiasis |  |  |  |  |
| - Treatment of intestinal worms |  |  |  |  |

1. **Focus Group Discussion Guide for Community Members**

***PLEASE NOTE:*** *(1) Each sub-group should have between 5-8 members.*

*(2) Conduct 4 FGDs for each sub-group per study district*

**Instructions:**

1. **Who to interview:**
   1. Youth male
   2. Youth female
   3. Adult male
   4. Adult female
2. **Sample:** 4 groups of 5-8 persons for each category (youth male, youth female, adult male and adult female) per study District.
3. **When:** Administer instrument before and after intervention

**Introduction**

Good day, I am ________________ and my colleagues are _________________ from the Ministry of Health and are here to learn from you about some type of activities related to the delivery of health interventions such as schistosomiasis and intestinal worms treatment that are going on in this community. We have invited you because of your experience in this community and the confidence we have in you.

Please note that in this discussion there are no right and wrong answers. Every opinion is important and please feel free to express yourself. What we will learn from you today will be useful in the future to implement MDA activities.

We will talk to you for about 45-60 minutes. Participation in this interview is voluntary and you may choose to terminate the interview if you decide without any repercussion. What you tell us will help us develop a strategy for training community members to manage the interventions stated earlier and thus improve the health of the people in this community. Your name and what you say to us for this study will be kept private. We wish to request your permission to take notes and tape record the conversation so as to ensure that we do not miss out or misrepresent any of your views after the discussion.

Do you have any questions about the study? In case you do have questions in the course of the discussion or afterwards, please do not hesitate to contact XXXXX or the National Health Sciences Research Committee on XXXXX.

Are you willing to participate? Yes [__] No [__]

**If no, thank participants and terminate discussion.**

**General information**

1. What are the healthcare facilities serving this community within this area?

Probe into:

- Availability of healthcare facilities and how they function in the community
- Who owns the facilities
- Who manages them
- Who uses the facilities
- Nearness of the facilities
- How well are these healthcare facilities satisfying the need of this community
- Existence of Village Health care Workers and type of activities they perform and at what cost? Gender distribution? Why?

1. What types of diseases do you experience in this community?

Probe for:

- Most common diseases experienced in this community
- The most serious and severe of these diseases to the community members? Please explain.
- Existence of some relationship between community members and the following diseases: schistosomiasis and intestinal worms? What is the community’s perception of schistosomiasis and intestinal worms? Does the community see it as a problem? Who are the risk groups? Why?
- Reasons for such relationships.

**Community participation and decision-making in health services delivery**

**Introduction:** *The experiences of using health workers in managing and controlling diseases that exist in many countries are costly and services may not be easily available. It has been shown that since these diseases are within the community, therefore, the community could take charge of managing and controlling these diseases through active participation. MDA strategy has been successful and we would like to see if this approach could be used in delivery of MDA for the treatment and control of schistosomiasis and intestinal worms in this community.*

1. What are your perceptions and attitudes towards delivery of health services using community based mode of delivery?
2. What are the roles of community members, gender, minority and different social political groups in delivery of community based health services? Please explain.
3. When were MDA campaigns introduced in this community?

Probe into:

- The people who brought the idea about MDA campaigns
- Whether there was a community meeting at the time
- Number of people selected and trained to be community volunteers
- How did MDA campaign volunteer selection relate to existing health activities?

1. How are the MDA campaigns performing in this community?

Probe into:

- Number of MDA campaign volunteers still functioning (if some of the volunteers are no longer functioning, probe for reasons why?)
- Method of incentive for the volunteers? Who provides the incentives?
- Ask about the mix of interventions – how are they working together, how satisfied are people and implementers?

1. Please describe how the community took the decision of implementing the MDA campaigns during the past years?

Elicit specific decisions/actions and then probe to the following issues:

- People involved in the decision making
- How the people in the community came to know the decisions?
- Method and time of distribution of drugs
- Have they participated and approved the volunteers’ selection?
- How does the community reward community volunteers? Probe for the decision process for incentives, if any?

1. In what ways can members of the community support the delivery of health services at community level?

Probe for:

- Community participation in delivery of health interventions at community level
- Community contribution towards delivery of community based health services
- Community members perception of use of incentives in delivery of community based health services
- Perceived opportunities, challenges and synergies for use of community based approach for delivery of health services

**End of the discussion. Please thank the participants for their time and participation.**
